# Modeling HER2 Effects on Cell Behavior from Mass Spectrometry Phosphotyrosine Data

**DOI:** 10.1371/journal.pcbi.0030004

**Published:** 2007-01-05

**Authors:** Neil Kumar, Alejandro Wolf-Yadlin, Forest M White, Douglas A Lauffenburger

**Affiliations:** 1 Department of Chemical Engineering, Massachusetts Institute of Technology, Cambridge, Massachusetts, United States of America; 2 Department of Biological Engineering, Massachusetts Institute of Technology, Cambridge, Massachusetts, United States of America; 3 Center for Cancer Research, Massachusetts Institute of Technology, Cambridge, Massachusetts, United States of America; University of California San Diego, United States of America

## Abstract

Cellular behavior in response to stimulatory cues is governed by information encoded within a complex intracellular signaling network. An understanding of how phenotype is determined requires the distributed characterization of signaling processes (e.g*.*, phosphorylation states and kinase activities) in parallel with measures of resulting cell function. We previously applied quantitative mass spectrometry methods to characterize the dynamics of tyrosine phosphorylation in human mammary epithelial cells with varying human epidermal growth factor receptor 2 (HER2) expression levels after treatment with epidermal growth factor (EGF) or heregulin (HRG). We sought to identify potential mechanisms by which changes in tyrosine phosphorylation govern changes in cell migration or proliferation, two behaviors that we measured in the same cell system. Here, we describe the use of a computational linear mapping technique, partial least squares regression (PLSR), to detail and characterize signaling mechanisms responsible for HER2-mediated effects on migration and proliferation. PLSR model analysis via principal component inner products identified phosphotyrosine signals most strongly associated with control of migration and proliferation, as HER2 expression or ligand treatment were individually varied. Inspection of these signals revealed both previously identified and novel pathways that correlate with cell behavior. Furthermore, we isolated elements of the signaling network that differentially give rise to migration and proliferation. Finally, model analysis identified nine especially informative phosphorylation sites on six proteins that recapitulated the predictive capability of the full model. A model based on these nine sites and trained solely on data from a low HER2-expressing cell line a priori predicted migration and proliferation in a HER2-overexpressing cell line. We identify the nine signals as a “network gauge,” meaning that when interrogated together and integrated according to the quantitative rules of the model, these signals capture information content in the network sufficiently to predict cell migration and proliferation under diverse ligand treatments and receptor expression levels. Examination of the network gauge in the context of previous literature indicates that endocytosis and activation of phosphoinositide 3-kinase (PI3K)-mediated pathways together represent particularly strong loci for the integration of the multiple pathways mediating HER2′s control of mammary epithelial cell proliferation and migration. Thus, a PLSR modeling approach reveals critical signaling processes regulating HER2-mediated cell behavior.

## Author Summary

Cells in the human body interpret extracellular information to “decide” on the execution of particular behaviors such as migration, proliferation, and differentiation. Many diseases, such as cancer, occur when these decision-making processes are compromised. The transfer of extracellular information to the intracellular space is often accomplished through receptor proteins whose chemical properties are altered as extracellular conditions change. These receptors transfer information in the intracellular space through the transfer of phosphate groups from one molecule to another. In particular, the transfer of phosphate groups to tyrosine sites is critical for cellular signaling. How the cell decides to execute a particular behavior on the basis of many changing phosphorylation events, however, is not understood. Here, we apply a computational approach to understand and predict how cells make the decision to migrate and proliferate as extracellular information changes. In particular, we wanted to understand the basis of decision-making processes in cells overexpressing a receptor protein called human epidermal growth factor receptor 2 (HER2). This receptor is overexpressed in ∼30% of breast cancer patients and correlates with poor prognosis. Taking advantage of a recently published dataset that quantified tyrosine phosphorylation events in HER2-overexpressing cells, we created models to understand and to predict HER2-mediated changes in migration and proliferation. The model identified small subsets of measured phosphorylation events that are predictive of changes in behavior with HER2 overexpression. Analysis of the phosphorylated subset of proteins implicated certain cellular processes as being crucial for cellular decision making, and suggested potential biomarkers and targets for therapeutic use in HER2-overexpressing cancers. Further application of our technique should aid in the understanding of cellular decision processes from large sets of cell signal and behavior data.

## Introduction

Recent advances in mass spectrometry have enabled the extensive characterization of intracellular signaling networks [1,2]. Coupled with the increasing appreciation that cell behavior is governed by a network of signaling events, these advances have been used to identify novel elements of network activation giving rise to cell behavior. Identification of such elements in the past has largely been accomplished in a nonstructured way through the manual parallel comparison of fold-change phosphorylation and cell phenotype [3,4]. We sought to use a mathematical formalism based on linear mapping to draw predictive connections between cell behavior (migration and proliferation) and a mass spectrometry dataset describing changes in intracellular tyrosine phosphorylation as human epidermal growth factor receptor 2 (HER2) was overexpressed under a variety of ligand treatment conditions.

HER2, a member of the ErbB family of receptors, is overexpressed in ∼30% of breast cancer patients and correlates with poor prognosis and high invasiveness [5]. Other members of the ErbB receptor family include epidermal growth factor receptor (EGFR), human epidermal growth factor receptor 3 (HER3), and HER4. These receptors give rise to one of the most extensively studied signaling networks in biology through a variety of ligand binding and dimerization schemes [6,7]. Epidermal growth factor (EGF) and heregulin (HRG), two ErbB family ligands, have been shown to induce both proliferation and migration to varying extents in breast cancer cells, although the signaling mechanisms responsible for this are not fully understood [8,9]. EGF predominantly binds EGFR to induce both EGFR homodimers and EGFR–HER2 heterodimers, whereas HRG predominantly binds HER3 and HER4, inducing HER2–HER3 and HER2–HER4 heterodimers. To obtain a dynamic and quantitative description of intracellular signaling in response to treatment with EGF or HRG and changing HER2 levels, we employed a mass spectrometry approach that measured levels of tyrosine phosphorylation on both receptors and other intracellular signaling molecules. Cell migration and proliferation were also quantified under the same treatment conditions [10]. Partial least squares regression (PLSR), a technique previously shown to be useful for the creation of signal–response models based on highly dimensional datasets, was used to correlate phosphorylation events to both migration and proliferation [10].

In this study, we significantly extend our previous analysis of HER2-mediated signaling and cell function by using a PLSR model to identify a reduced set of phosphorylation measurements and computational rules that together can be used to predict a priori cell migration and proliferation. We also characterize ligand-specific changes in cell signaling that govern migration and proliferation through the novel application of inner product analysis. Specifically, we derive lists of the most important phenotypically relevant proteins characterizing each of 30 possible transitions between our six cell conditions (EGF, HRG, and serum-free treatments in both low and high HER2-expressing cells). Inspection of the lists reveals both regulatory signaling cascades consistent with known HER2 biology and novel hypotheses. Using a conceptually similar procedure, we also derived lists of proteins that uniquely correlated with either migration or proliferation, postulating that these proteins serve as migration- or proliferation-specific signals in our system. Finally, we analyzed the PLSR model to derive a subset of phosphorylation sites most informative for the quantitative prediction of migration and proliferation. We identified nine phosphosites (signals) on six proteins from the original 62 phosphosites (signals), and showed that a model based on only those nine sites had a goodness of fit to experimental data similar to the full model. We identify the nine signals as a “network gauge,” a subset of molecules in the vast network of signaling molecules that together serve as a sensitive readout for cellular response. The nonobvious nature of the nine selected signals highlights the complexity of the network and the usefulness of the modeling approach. Analysis of the network gauge suggests that two elements of network architecture, endocytosis and phosphoinositide 3-kinase (PI3K)-related signaling, are highly informative loci for the control of proliferation and migration. Importantly, models constructed from both the full and network gauge signaling data that were trained only on data from a low HER2-expressing cell line predicted levels of migration and proliferation in a HER2-overexpressing cell line for both EGF and HRG treatments. This suggests that both cell types process information in the signaling network according to the same set of multilinear rules.

## Results

### Mass Spectrometry Approach Measures 62 Intracellular Signals in Human Mammary Epithelial Cells

**Figure 1:**
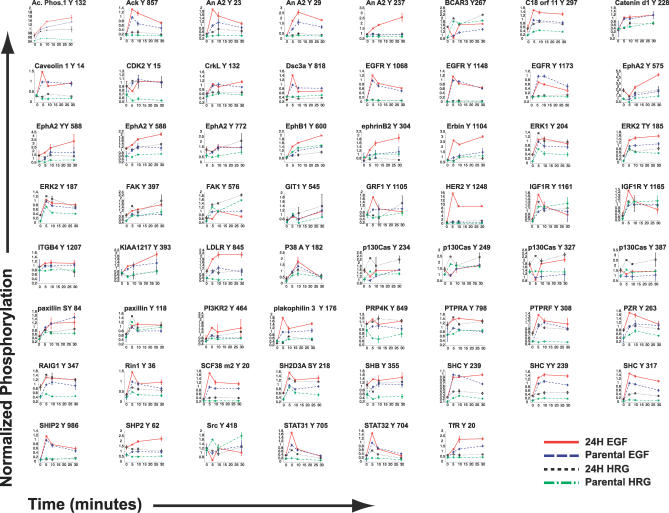
A Mass Spectrometry Approach Measured 248 Phosphorylation Profiles The title at the top of each entry indicates the phosphosite measured. Median normalized phosphorylation (see Methods) is plotted at 0, 5, 10, and 30 min. For each phospho-site, four conditions were measured: parental + HRG (80 ng/ml), 24H + HRG (80 ng/ml), parental + EGF (100 ng /ml), and 24H + EGF (100 ng/ml). Error bars indicate ± standard deviation. The data were obtained from [10].

As previously described, we developed and employed a mass spectrometry approach to measure the effect of HER2 overexpression in 184A1 human mammary epithelial cells (HMEC) [10]. Two closely related cell lines with known receptor expression levels were used; a parental cell line (P) with 200,000 EGFR, 20,000 HER2, and 20,000 HER3, and a HER2-overexpressing cell line (24H) with 200,000 EGFR, 600,000 HER2, and 30,000 HER3 per cell [10]. Both cell lines have very low levels of HER4. Thus, the 24H cell line was used to assess effects of HER2 overexpression, with the parental cell line serving as a baseline for these measurements. HMECs were treated with saturating levels of EGF or HRG, and under each treatment condition the tyrosine phosphorylation of 62 phosphosites was quantified at 0, 5, 10, and 30 min. Figure 1 displays the 248 time courses collected. Our measurements revealed the dynamic activation of molecules commonly associated with ErbB signaling (e.g., extracellular regulated kinase 1 [ERK1] and SH2-containing protein [Shc]) and others less commonly associated with the ErbB network (e.g., human transferrin receptor [TfR], ephrin A2 receptor [EphA2], and the previously unidentified KIAA 1217). Comparison with previously published maps of ErbB and migration-associated signaling networks reveals broad network coverage with the 62 measured signals [7,11].

### Cell Proliferation and Migration Are Differentially Affected by HER2

**Figure 2:**
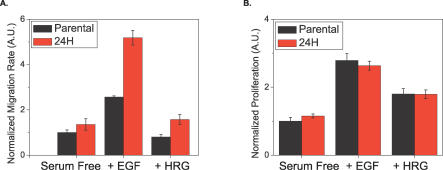
HER2 Overexpression Affects Migration but Not Proliferation Parental (black) and 24H (red) data shown for: (A) migration, as measured by a wound healing assay, and (B) proliferation, as measured by [3H] thymidine incorporation. Migration error bars represent the 95% confidence intervals for the fit of the slope using linear regression. Proliferation error bars represent the standard deviation from four different biological replicates for each condition. The data were obtained from [10].

Cellular migration and proliferation were measured under the same conditions described above [10]. HER2 overexpression correlates with increased cell migration under all ligand-stimulating conditions (Figure 2A). EGF treatment increased the rate of migration for both cell lines by the highest absolute amount, whereas HRG treatment did not increase migration as compared with the serum-free case in either cell line. In contrast to migration, proliferation was not significantly altered by HER2 overexpression (Figure 2B). Both HRG and EGF increased proliferation above serum-free levels, with EGF stimulating the highest absolute amount of proliferation [10].

### Model Analysis Reveals Phenotype-Relevant Signaling Sets That Characterize Ligand and Receptor Expression Transitions

A model based on PLSR was created to linearly regress signaling metrics onto cellular migration and proliferation metrics ([10] and Methods). The model accurately recapitulated experimental data and had a goodness of prediction (Q^2^) of 0.89 (Figure S1). Each signal comprised four metrics: the 5, 10, and 30 min phosphorylation levels, and the integral of the time course from 0 to 30 min. The integral was used as a measure for total phosphorylation. The 0-min time point was included in the row of serum-free observations (see Methods).

**Figure 3:**
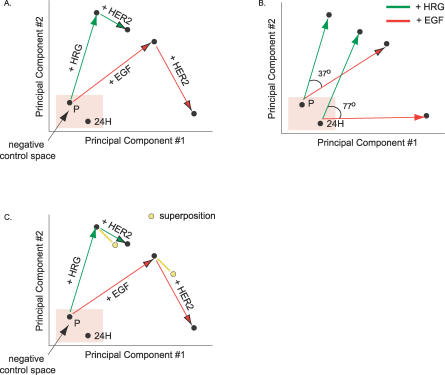
PLSR-Generated Scores Plot Reveals Different Signaling Strategies for EGF and HRG (A) A scores plot identifies separation in signaling strategies associated with receptor overexpression or differential ligand treatment along two signaling axes. (B) HRG and EGF treatment give rise to different sets of signals, and the difference is exaggerated in 24H cells. (C) The linear superposition of the difference vector between 24H and parental serum-free conditions and each ligand's vector explains 24H + HRG signaling but not 24H + EGF signaling.

Decomposition via PLSR of the signal (**X**) and response (**Y**) datasets provided a reduced-dimension map (called the scores vector **t**, see Methods) on which network signaling changes in response to ligand or receptor perturbation could be interpreted. The plot axes (referred to as principal components) are linear combinations of the signaling metrics described above. Figure 3 plots the changes corresponding to ligand or receptor perturbation on a two-dimensional graph whose axes are the first two principal components (the third component, which captures only 4% of the **Y**-block variance described by the full model, is omitted here for ease of visualization). The plot shows, as expected, that HRG treatment stimulates the signaling network distinctly from the EGF treatment case. If HRG treatment activated the same set of signals as EGF with different magnitudes, we would observe the +EGF and +HRG vectors overlapping, with one being longer than the other. Since HRG and EGF drive the formation of different HER dimers (i.e., HER3–HER2 versus EGFR–HER2 or EGFR–EGFR), we expect differential activation of the signaling network. Interestingly, the difference between EGF and HRG signaling is larger in 24H cells relative to parental cells as indicated by the offset of the two vectors (77 degrees versus 37 degrees, respectively), due in large part to a drastic shift in EGF-induced signaling with HER2 overexpression (Figure 3B). If we linearly superpose changes in signaling due to HER2 overexpression in the absence of ligand (i.e., the signaling changes under serum-free conditions between P and 24H cells) and the changes in signals as we add a ligand (i.e., signaling changes as either HRG or EGF are added to P cells), we can approximate the signals generated by 24H cells under HRG treatment (Figure 3C). We cannot do the same, however, for 24H cells under EGF treatment, emphasizing the nonadditive interplay between changes in cellular ligand–receptor conditions and the signals they generate (Figure 3C). In the case of our HMEC system, previous quantitative measurement and modeling of dimer kinetics has shown that HER2 overexpression in the presence of HRG leads to a relatively small shift in the number of HER2–HER3 dimers (∼10,000). Alternatively, HER2 overexpression in the presence of EGF leads to a large increase in EGFR–HER2 dimers (∼150,000) and a decrease in EGFR homodimers (∼65,000) [10]. Changes in signaling with HER2 overexpression under serum-free conditions could be due to either spontaneous homodimerization or autocrine signaling. Thus, given our analysis of the scores plot, we hypothesize that increases in HER2 under HRG treatment principally add to the signaling network through the independent addition of signals generated through HER2 homodimerization or autocrine signaling. HER2 overexpression under EGF treatment, however, results in the addition of these signals plus a novel suite of signals generated primarily through an increase in EGFR–HER2 dimers and loss of EGFR homodimers. These data support a previous finding that increasing levels of phosphorylated EGFR and HER2 induce novel signaling elements associated with lower affinity intracellular receptor–protein binding [12].

**Figure 4:**
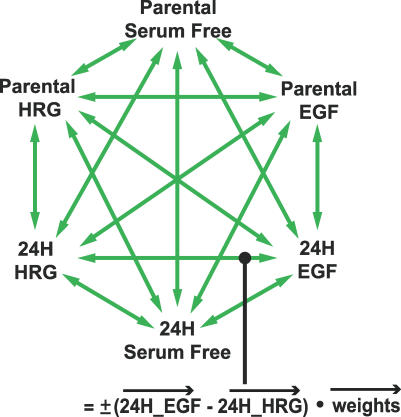
A Strategy for the Study of 30 Possible Cellular Transitions Each arrow represents the difference vector between two cell states. The changes in the signaling network associated with the transition between the two states are calculated by taking the inner product of the difference vector with the weights vector (see Methods).

Although the scores plots allow us to visualize signaling changes, it is often of interest to relate observed differences back to original measured signaling metrics. We accomplish this by taking the inner product of any vector in the scores plot with the principal component axes, and thereby derive lists of proteins that most strongly correlate with the transition associated with the vector (see Methods). Figure 4 outlines this approach and indicates the 30 cell state transitions analyzed. We discuss here a subset of those transitions, but all results are available in Dataset S1.

HER2 overexpression in the presence of EGF, as discussed above, produced interesting signal network changes and increased cell migration but did not affect cell proliferation (see Figure 2). Using our approach, we identified proteins correlating most positively with HER2-associated increases in motility under EGF treatment (Table 1). HER2 phosphorylation at tyrosine 1248 and that of Crk-associated substrate (p130Cas) at tyrosine 327 feature prominently in the list, agreeing with previous reports linking the HER2-specific activation of p130Cas to increased invasion in breast epithelial cells [9]. Another protein listed is annexin A2 (also known as lipocortin 2), a molecule previously known to mediate cytoskeletal–membrane interactions, therefore linking it to critical processes governing cell migration [13]. While expression of annexin A2 has been found to decrease or increase cell migration, depending on the cell system, little is known about how its phosphorylation correlates with cell migration [14,15]. Here, we speculate that annexin A2 is part of the mechanism that increases cell migration under HER2 overexpression in the presence of EGF, and we identify a novel phoshorylation site, tyrosine 237, that may regulate its role in the activation of migration. Phosphorylation of the SH2 domain–containing phosphatase SHP-2, another molecule that appears in Table 1 multiple times, has been shown to increase cell migration in breast cancer cells, although connection to particular phosphorylation events has been sparse [16]. Here we implicate the tyrosine 62 site in SHP-2′s activation and eventual effect on cell migration. Interestingly, SHP-2 and annexin A2 have been found to complex in endothelial cells, suggesting the possible presence of a co-regulatory scheme in our HMEC system [17].

**Table 1:**
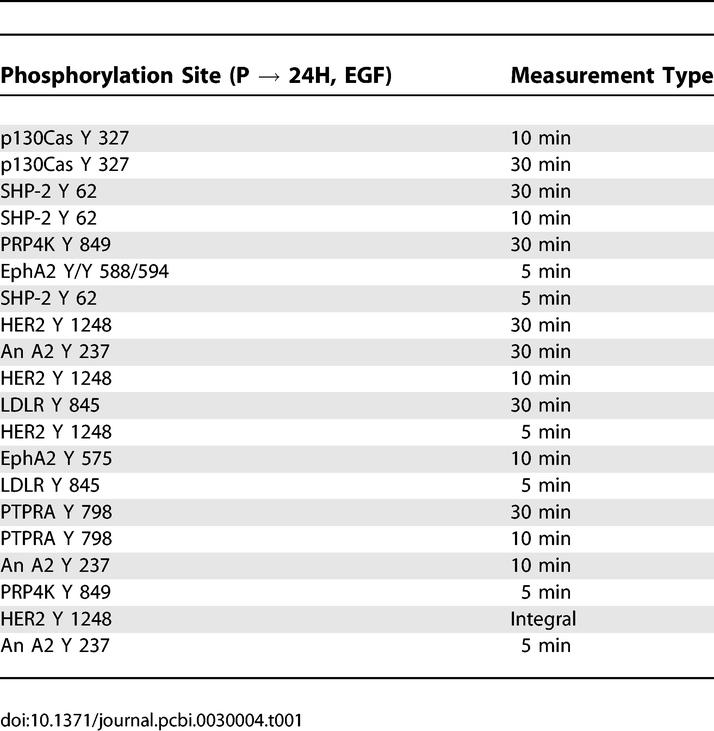
Signaling Metrics Most Positively Correlated with Changes in Cellular Response due to Increased HER2 Expression under EGF Stimulation

A list of proteins most negatively correlated with phenotype includes all measurements of EGFR phosphorylated at tyrosine 1173 as well as Src, which has been shown to phosphorylate EGFR tyrosine 1173 (Table 2, [18]). These molecules exhibit decreased phosphorylation in response to increasing HER2 expression and concomitant increases in migration under EGF treatment. The tyrosine 1173 site on EGFR has been shown to recruit SH2 domain–containing phosphatase SHP-1, which helps coordinate EGFR dephosphorylation and mitogen-activated protein kinase deactivation [19]. We speculate that decreased tyrosine 1173 phosphorylation is part of the mechanism through which HER2 increases the downstream signaling governing increased migration.

**Table 2:**
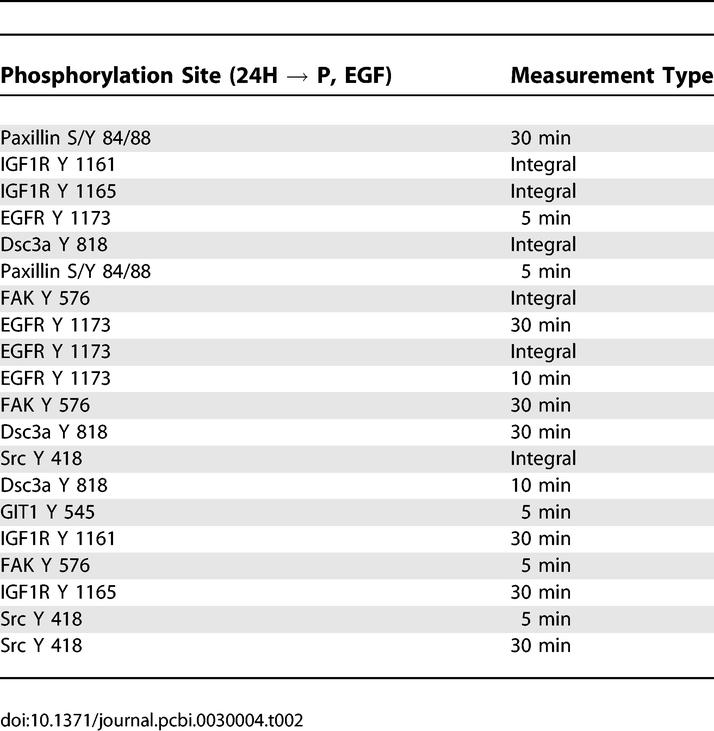
Signaling Metrics Most Negatively Correlated with Changes in Cellular Response due to Increased HER2 Expression under EGF Stimulation

Next, we sought to understand the effect of changing ligand under given receptor expression levels (Tables 3 and 4). Substitution of EGF for HRG with 24H cells increases both proliferation and migration, although the absolute increase in migration is greater (Figure 2). Interestingly, many proteins that negatively correlate with this transition are linked to the migration-relevant p130Cas pathway (Table 4). This pathway includes Src and its substrates focal adhesion kinase (FAK) and p130 Cas [FAK also phosphorylates p130Cas] [20,21]. Counterintuitively, then, EGF appears to negatively regulate a classical migration pathway to increase cell migration. The new EGF-stimulated signals not only increase migration but proliferation as well. In Table 3, we observed proteins previously linked in the literature to migration (e.g*.*, annexin A2, glucocorticoid receptor DNA binding factor 1 [GRF1]), and others linked to proliferation (EGFR, desmocollin-3 [Dsc3a]) [7,10,13]. KIAA 1217 is a previously unidentified protein that warrants further investigation for its potential role in EGF-mediated proliferation and migration.

**Table 3:**
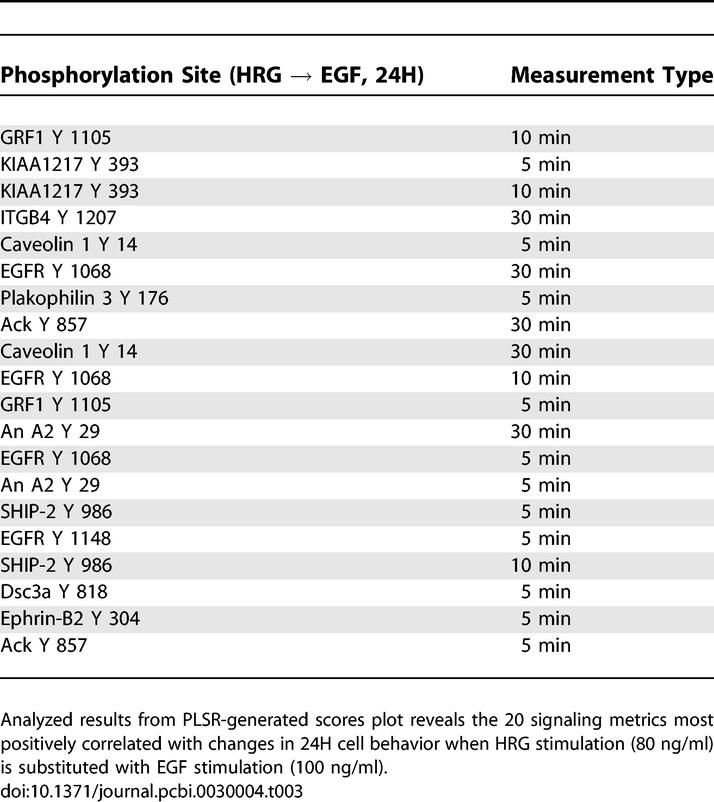
Signaling Metrics Most Positively Correlated with Changes in Cellular Response due to Varying Ligand Exposure in Cells with High HER2 Expression

**Table 4:**
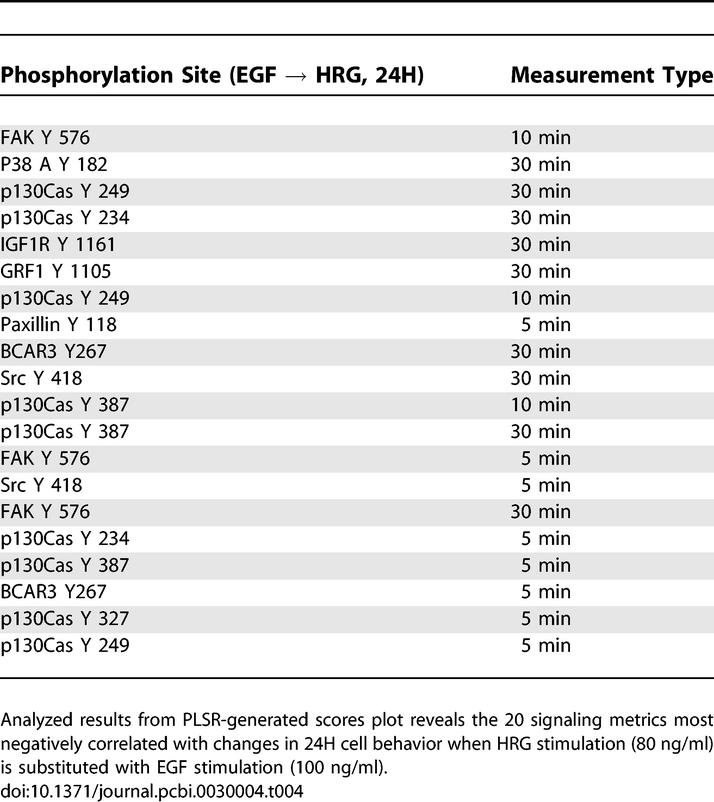
Signaling Metrics Most Negatively Correlated with Changes in Cellular Response due to Varying Ligand Exposure in Cells with High HER2 Expression

As mentioned above, the signaling changes associated with increased HER2 expression in the presence of HRG are very similar to the same change under serum-free conditions. In both cases, HER2 overexpression leads to an increase in migration but not proliferation. Signaling metrics that positively correlate with this transition include p130Cas and FAK, indicating that increased migration may be mediated through this migration-associated pathway (Tables 5 and S1). Serine/threonine protein kinase PRP4 homolog (PRP4K) and protein tyrosine phosphatase receptor type A (PTPRA) are two additional molecules that correlate with increased migration suggesting a novel role for both in HER2-mediated migration. Activation of these molecules, as mentioned above, may be due to spontaneous HER2 homodimerization or autocrine signaling. Considering Table 6, we note that phosphorylation of EGFR tyrosine 1173 negatively correlates with the increase in migration, just as we observed for HER2 overexpression in the presence of EGF. HRG treatment, however, is generally not thought to regulate EGFR phosphorylation, and the presence of tyrosine 1173 in Table 6 suggests that HER2 overexpression in the presence or absence of HRG drives migration through autocrine signaling that activates EGFR–HER2 heterodimers. Indeed, we have shown that there is a measurable but low amount of transforming growth factor alpha (TGF-α) produced by both the parental and 24H cells (personal communication, Lisa Joslin and Douglas Lauffenburger), thus pointing to a potential mechanism for autocrine-induced signaling changes.

**Table 5:**
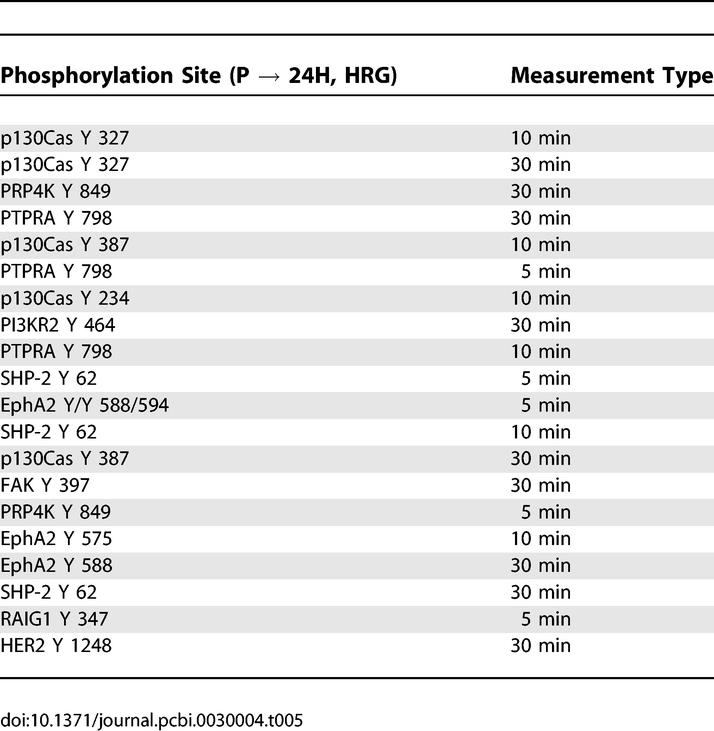
Signaling Metrics Most Positively Correlated with Changes in Cellular Response due to Increased HER2 Expression under HRG Stimulation

**Table 6:**
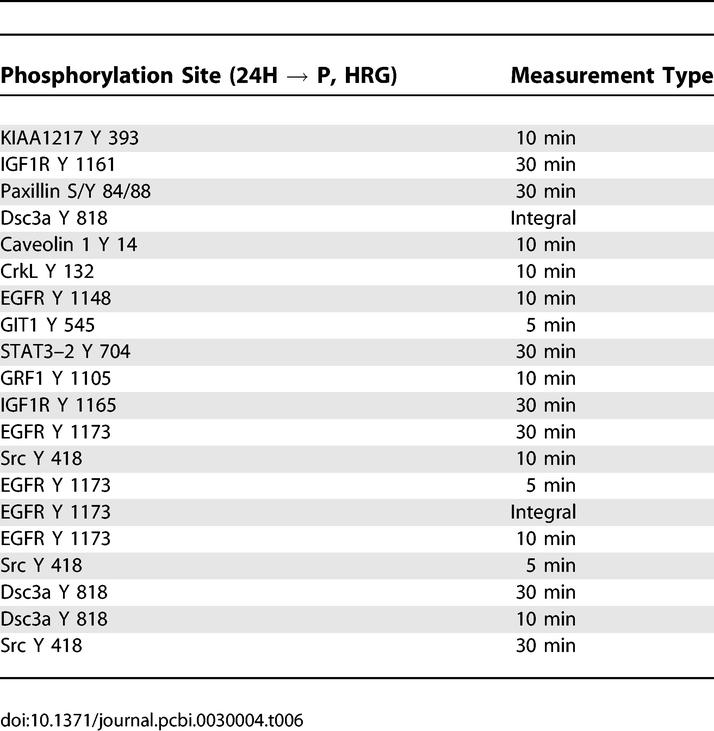
Signaling Metrics Most Negatively Correlated with Changes in Cellular Response due to Increased HER2 Expression under HRG Stimulation

### A Nine-Signal Reduced Model Recapitulates Full-Model Performance

**Figure 5:**
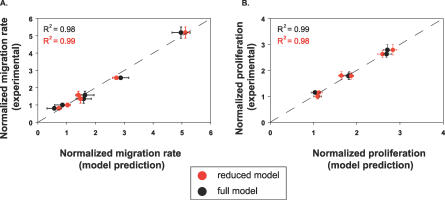
A Linear Model Based on Nine of the Original 62 Signals Recapitulates Experimental Data and Matches Full Model Performance Results from PLSR modeling show that a computational model based on six signals generates predicted values of cellular migration and proliferation that correlate strongly with those predicted by the full model and experimentally measured values. Experimental values of migration (A) or proliferation (B) are graphed along the ordinate, and full-model predictions (black) and reduced-model predictions (red) of migration (A) or proliferation (B) are given along the abscissa. R^2^ values indicate the data's goodness of fit to the line y = x. Experimental error bars denote 95% confidence intervals for migration (see Methods) and ± standard deviation for proliferation. All computational error bars are calculated from jack-knifing as a standard error.

A reduced model based on a fraction of the 62 originally measured phosphorylation sites would be useful for the future study of HER2 effects when full network measurement is not possible. Analysis of the model revealed nine phosphorylation sites on six proteins that recapitulated the performance of the full model. We refer to this subset of signals as a “network gauge”: a small number of phosphorylation sites that together can be interrogated to predict levels of proliferation and migration. To rank phosphorylation events according to their importance in the full model, we used a weighted sum of squares technique (see Methods). Phosphorylation sites whose metrics appeared more than once in a list of the top 30 weighted sum of squares metrics were selected for the reduced model. Using this method, phosphorylation sites on the following six proteins were identified as important: TfR, annexin A2, activated cdc42-associated kinase (ACK), SH2-containing inositol polyphosphate 5-phosphatase (SHIP-2), SH2-containing protein (Shc), and solute carrier protein 38 (SCF38, also known as SNAT2 or ATA2). All measured tyrosine phosphorylation sites were included for these molecules except for the tyrosine 237 site on annexin A2, since it was not represented at any time on the top 30 list. A model generated with this six-protein set had an excellent goodness of fit to experimental data compared with the full model (Figure 5). In addition, the reduced model maintained a high goodness of prediction (Q^2^ = 0.95). A model based on the bottom-30 ranked metrics had a goodness of fit less than 0.30 to experimental data, suggesting that our ranking appropriately isolated highly informative sets of phosphorylation metrics.

The somewhat surprising makeup of the network gauge prompted further investigation into why these six proteins were so informative. Shc's tyrosine site at 239/240 regulates c-myc activation; its tyrosine site at 317 regulates mitogen-activated protein kinase activation; and its phosphotyrosine-binding domain is known to associate with phosphatidylinositol-3,4,5-trisphosphate (PIP3), although it is not known how Shc's binding affinity for PIP3 changes with tyrosine phosphorylation [22]. Thus, tyrosine phosphorylation of Shc affects multiple important signaling pathways, leading us to speculate that the dynamic and quantitative measurement of Shc at both tyrosine phosphosites may serve as a sensitive indicator for the ultimate activation of pathways important for proliferation and migration. The aforementioned annexin A2, a target of kinases such as Src and protein kinase C, has been found to mediate membrane–cytoskeleton interactions, and its knockdown has been linked to decreased invasiveness in human glioma cell lines [23]. Annexin A2 clusters strongly with migration in the reduced model, revealing its role as a relatively migration-specific predictor. TfR endocytosis brings iron into the cell and is stimulated by tyrosine 20 phosphorylation [24]. Introduction of iron into the cell can be a major regulator of cell proliferation and growth, and TfR has also been linked to migration in endothelial cells [25,26]. Moreover, TfR has been shown to partially traffic with EGFR, although it may internalize via a different mechanism [27–29]. To our knowledge, this is the first report of EGF-stimulated TfR phosphorylation and regulation via HER2 expression, and we hypothesize that tyrosine phosphorylation of TfR is a reliable indicator of endocytic regulation in our system. ACK, downstream of cdc42, has been shown to bind clathrin and to regulate TfR trafficking, implicating it also as a part of the endocytosis readout [30]. ACK has additionally been shown to be an early transducer of EGFR signaling and to negatively regulate cell adhesion through the CrkII-p130Cas pathway, a pathway shown above to be important in HER2-mediated migration [9,31,32]. SHIP-2 is a phosphatase that acts on PIP3 and has been found associated with filamin and actin, implicating it directly in the regulation of both PI3K signaling pathways and cell migration [33]. Additionally, SHIP-2 regulates EGFR trafficking and associates with Shc in response to EGF binding, further suggesting that SHIP-2 phosphorylation is a sensitive readout for a wide variety of different signaling responses [34,35]. SCF38 is a System A amino acid transporter that responds to growth factor signaling [36]. Insulin stimulation leads to the recruitment of SCF38 to the cell membrane through a PI3K-dependent signaling mechanism that coordinates its trafficking from the endosomal compartments [37]. Little is known about potential roles for SCF38 in ErbB signaling, but based on our model results, further biochemical studies are warranted.

Two interesting themes, then, emerge from the network gauge findings: (a) the inclusion of a group of molecules linked to endocytosis, namely TfR, ACK, and SHIP-2; and (b) the high proportion of molecules known to interact with PI3K or PIP3, namely Shc, SHIP-2, TfR, and SCF38. We will elaborate further on these themes in the Discussion section.

### Models Based on Parental Cell Data Alone Accurately Predict the Effects of HER2 Overexpression on Proliferation and Migration

**Figure 6:**
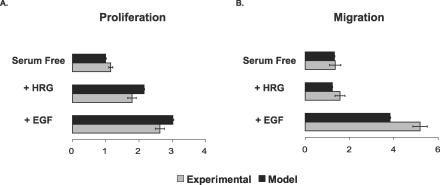
A Reduced-Signal PLSR Model Based Only on Parental Cell Data Predicts 24H Proliferation and Migration A PLSR model of nine signals constructed from parental cell data only was used to predict (A) proliferation and (B) migration levels in 24H cells and then compared with measured experimental values. Experimental error is denoted by 95% confidence intervals for cell migration (see Methods) and ± standard deviation for cell proliferation. Computational error bars were calculated as the 95% confidence intervals based on jack-knifing.

We investigated whether the full model and the network gauge trained only on parental cell data could predict migration and proliferation levels in response to HER2 overexpression. We trained models on parental serum-free, EGF, and HRG data, performed PLSR to calculate regression coefficients, and then used the measured 24H signal values in the regression equation to predict proliferation and migration. We found that both the full 62-signal model and the network gauge were able to predict proliferation and migration in 24H cells (R ≥ 0.99, Figure 6, Figure S2). Although the model does not match the migration or proliferation results exactly in terms of absolute quantities, it captures salient trends such as the sharp increase in migration under EGF treatment for 24H cells and the trend of increasing proliferation when HRG is substituted with EGF. Interestingly, the model also predicts a slightly decreased amount of migration under HRG stimulation in comparison with the serum-free condition, which corresponds to the lack of HRG-mediated migration observed experimentally. The predictive capability of the model indicates that although HER2 overexpression changes intracellular signals drastically, the rules by which those signals are brought together to affect proliferation and migration, as defined by PLSR, remain the same as in the parental cells.

### PLSR Analysis Reveals Signals That Uniquely Correlate with Migration and Proliferation

Identification of molecules highly, but uniquely, associated with either proliferation or migration are of value when considering strategies to inhibit one behavior without affecting the other. We previously reported the top 20 signals associated with migration and proliferation through an analysis of reduced-dimension PLSR plots [10]. Interestingly, however, we noted that most signals having a high projection for one output have a nonzero projection for the second output. Thus, the importance of a signal with respect to cellular phenotype is a quantitative assessment, and we can derive behavior-specific protein metrics by ranking the inner product of the metric and the relevant behavior after the inner product of that same metric with the second behavior has been subtracted (Methods). Tables 7 and 8 list the phosphorylation sites that most strongly and uniquely correlate with migration and proliferation, respectively. Table 7 indicates that attractive migration-specific targets include annexin A2 237 and HER2 1248 tyrosine phosphorylation. Likewise, Table 8 indicates that Dsc3a or catenin-δ1 are good targets for proliferation-specific inhibition. If the goal is to perturb one behavior without perturbing the other at all, then the corresponding protein targets could be calculated by making a list of proteins that had close to zero projection along the output one desired not to affect. The problem with this strategy is that the projection along the behavior one desires to affect may be small as well.

**Table 7:**
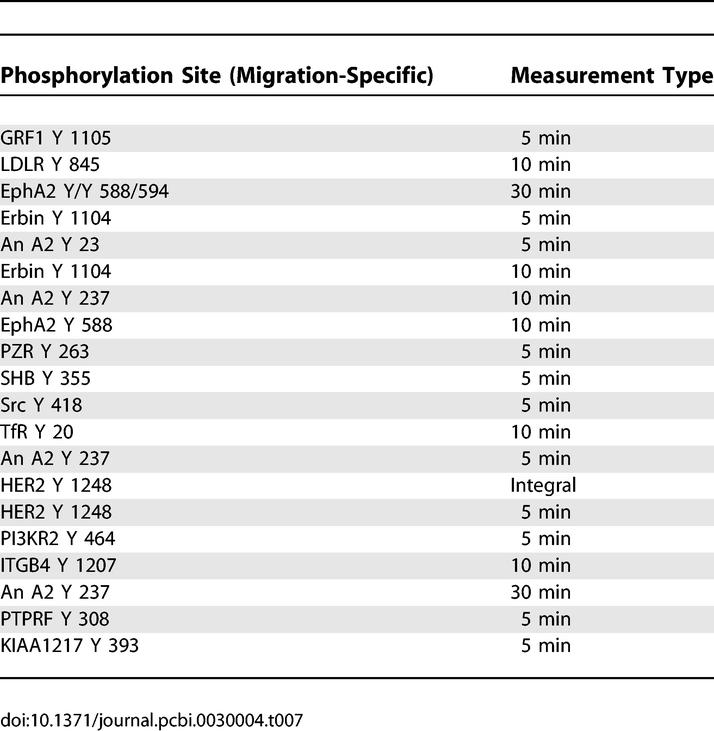
Analysis Results of PLSR X-Y Loadings Plot Reveals the 20 Signaling Metrics Most Uniquely Correlated with Migration

**Table 8:**
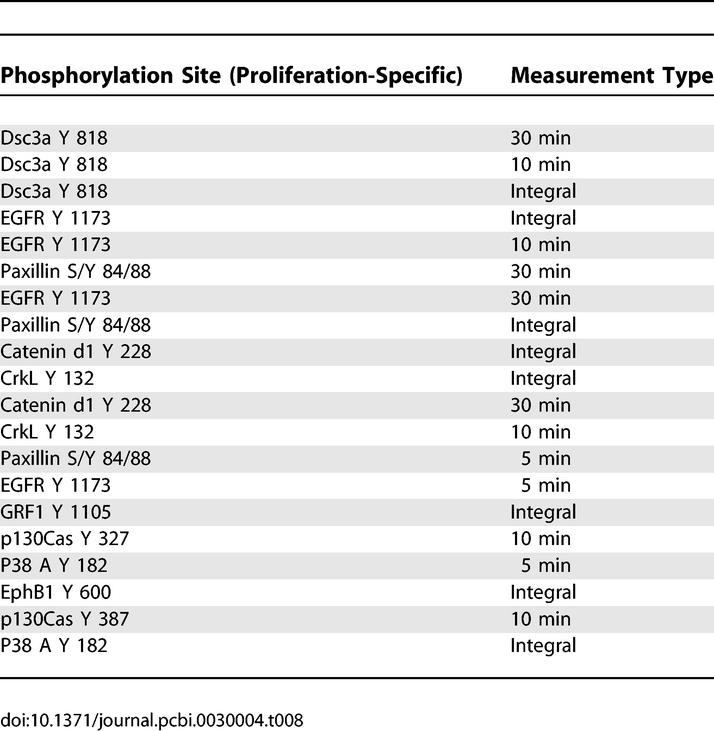
Analysis Results of PLSR X-Y Loadings Plot Reveals the 20 Signaling Metrics Most Uniquely Correlated with Proliferation

Table 9 lists proteins that positively correlate with both outputs to the greatest degree (i.e*.*, the sum of the inner products with both migration and proliferation). While there exists some redundancy with the migration and proliferation lists we published earlier ([10]), as expected, novel proteins such as p130Cas, FAK, and PRP4K have an elevated importance when their effects on migration and proliferation are summed. Although p130Cas is usually associated with control of invasion and cell motility in HER2–overexpressing breast epithelial cells, recent reports link it to proliferative control in HER2-dependent mammary epithelial tumorigenesis [38]. FAK phosphorylated at the tyrosine 576 site also appears on the list while being absent from the individual migration or proliferation lists. FAK regulates breast cancer cell migration and invasion, and also plays a role in cell cycle and proliferative control, although evidence for the latter role has been sparse in HER2-overexpressing systems [39–41]. Here, we hypothesize that FAK plays a critical role in the control of both proliferation and migration in the HMEC cell line, and taken together with the appearance of p130Cas and Src, we hypothesize that the well-described Src–FAK–Cas pathways play an essential role in the control of both proliferation and migration in HMEC cells. This activity could be captured by our network gauge in numerous ways. For instance, information about Src activity may be included via annexin A2 phosphorylation levels.

**Table 9:**
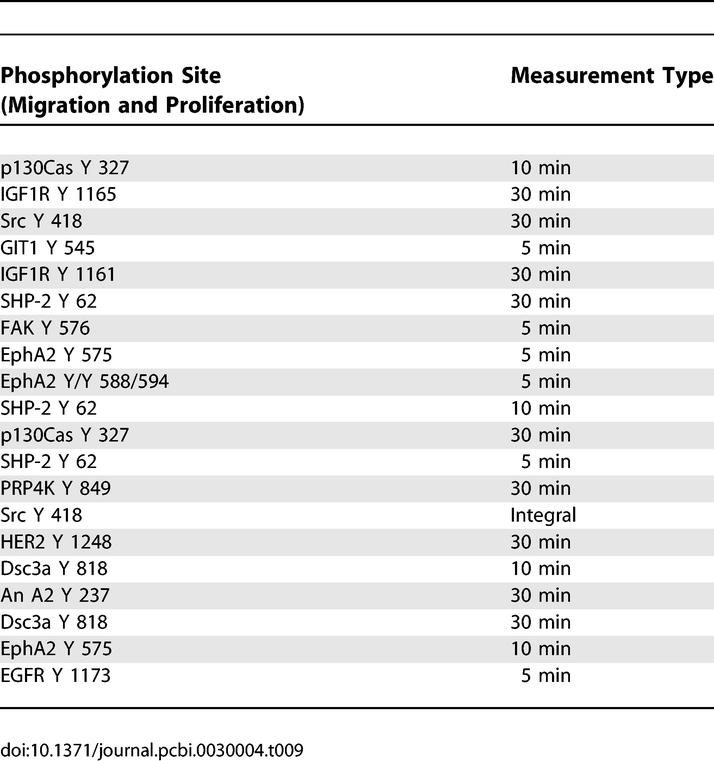
Analysis Results of PLSR X-Y Loadings Plot Reveals the 20 Signaling Metrics Correlated Most Strongly with Both Migration and Proliferation

Table 10 lists the 20 proteins that demonstrated weakest correlation with both migration and proliferation (i.e*.*, the sum of their projections along both behaviors were small). The list is of some interest for two reasons: (a) some of the proteins are also listed in Tables 7–9, but at different times or at different phosphorylation sites (i.e*.,* FAK or p130Cas), indicating the need for phospho-specific, time-resolved data; and (b) the presence of some proteins presumed to be of substantial relevance to HER2- associated signaling, such as ERK. The reason ERK is not important for the model here is that its variance upon receptor or ligand perturbation does not consistently linearly correlate with consequent proliferation or migration measures across all conditions. Nonetheless, we have observed that treatment with mitogen-activated protein extracellular kinase inhibitor PD98059 has an effect on the proliferation of HMEC cells (unpublished observations). This raises an important caveat concerning the model, namely that there can be proteins important for cell responses that do not consistently correlate across all or most treatment conditions. We do not claim to be able to identify all of the false negatives (or positives), but rather state that a model of this type remains predictive and enabling of conceptual insights.

**Table 10:**
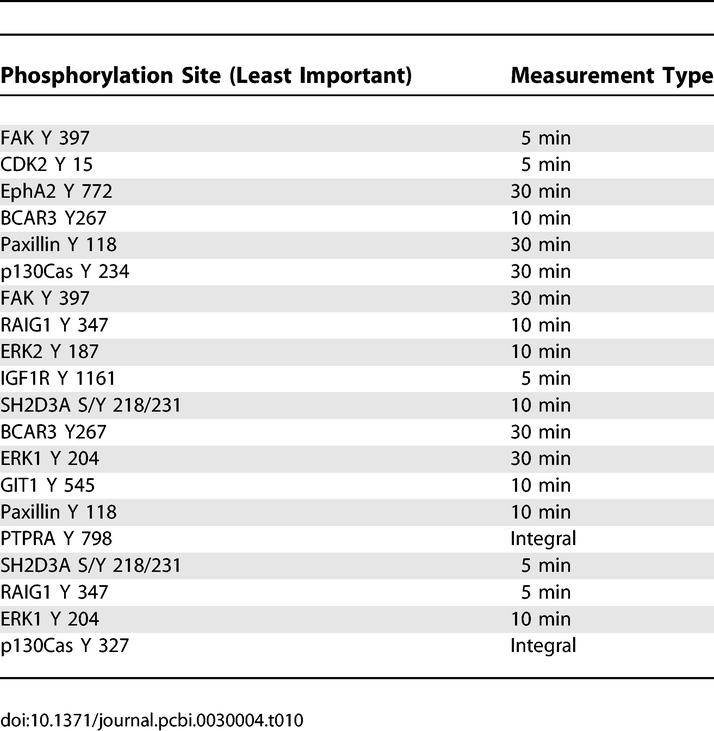
Analysis Results of PLSR X-Y Loadings Plot Reveals the 20 Least Correlated Signaling Metrics for Both Migration and Proliferation

## Discussion

**Figure 7:**
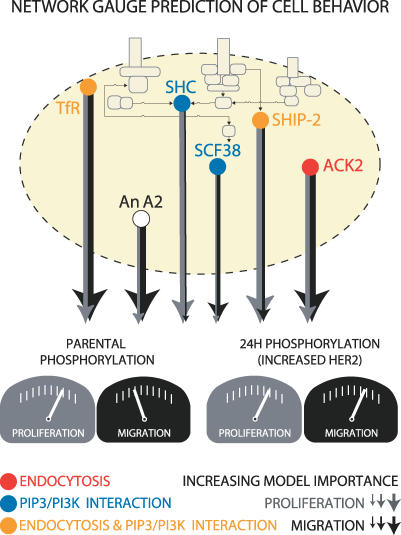
A Network Gauge Predicts Cell Behavior and Suggests Critical Elements of Network Architecture A nine-signal PLSR model trained on parental data predicts migration and proliferation in 24H cells. The line thickness emerging from each protein indicates the relative average importance of each protein in the model for migration (black) or proliferation (gray). Prediction of migration or proliferation is a function of model importance and the amount of phosphorylation measured in either parental cells or 24H cells. The proteins constituting the model are transferrin receptor (TfR), annexin A2 (An A2), solute carrier protein 38 (SCF38), SH2-containing protein (Shc), SH2-containing inositol polyphosphate 5-phosphatase (SHIP-2), and activated cdc42-associated kinase 2 (ACK2). Previously documented associations with endocytosis (red circle), PIP3/PI3K signaling (blue circle), or both (yellow circle) are shown. The absence of association with either endocytosis or PIP3/PI3K signaling is denoted by a white circle (An A2).

We have demonstrated the use of PLSR to characterize the relative importance of tyrosine phosphoryation events for cell migration and proliferation in two human mammary epithelial cell lines with varying HER2 expression levels under both EGF and HRG treatment. In addition, we have identified an important subset of molecules from our original large signaling dataset to serve as a network gauge for the prediction of migration and proliferation (Figure 7). Our results both highlight previously identified elements in the HER2 signaling network, and suggest new pathways and targets critically implicated in HER2-mediated signaling and its effect on migration and proliferation.

Scores plot analysis (Figure 3) helped generate global intuition as to how different combinations of ligand and receptor expression activated the phosphotyrosine signaling network. We related these changes back to original measurements through the use of inner products, generating lists of proteins correlated with any given ligand or receptor transition. Because the lists are derived after applying PLSR, the proteins highlighted have already been identified as important for the description of changes in cellular behavior. This procedure represents an improvement over traditional analysis of large mass spectrometry datasets (usually fold-change analysis) and demonstrates, to our knowledge, the first time an approach based on inner products has been used to extract understanding from PLSR-based biological models. Our lists (Tables 1–6) show that a particular behavior may be controlled through different network signaling strategies depending on cellular input. For instance, when EGF treatment replaces HRG in 24H cells, migration is stimulated through a different set of molecules than are used to elevate migration when HER2 levels are increased.

The reduction of the mass spectrometry dataset to nine highly informative phosphorylation sites on six proteins suggests elements of network architecture that likely control migration and proliferation, namely endocytosis and signaling through PIP3- and PI3K-mediated pathways. Three of the six highly informative proteins, TfR, SHIP-2, and ACK, are all linked to endocytosis [24,30,35]. The tight connection between endocytic regulation and the signaling networks governing cell migration and proliferation has been documented, most powerfully in a recent study using RNA interference against the human kinome [42]. The results of this study indicate that more kinases than previously appreciated are involved in endocytosis, and taken together with other recent efforts implicate endocytosis as a high-level regulator and sensor of cell-signaling networks [42,43]. Endocytosis can occur via many different mechanisms, principally clarthrin-mediated endocytosis and caveolar/raft-mediated endocytosis, with each mechanism regulating different sets of kinases and cell behaviors [42,43]. The fact that TfR endocytosis was identified as highly informative instead of EGFR endocytosis might be due to the fact that EGFR internalization is mediated by both clarthrin-mediated endocytosis and caveolar/raft-mediated endocytosis after treatment with high amounts of EGF, whereas TfR is thought to internalize independent from RCE [28]. The dynamic and quantitative resolution in our signaling assay was most likely critical for the capture of endocytic events, as endocytosis strongly regulates both signal duration and intensity. Furthermore, although our assay did not measure spatial distribution, endocytic information may have served as a proxy for that, further explaining its presence in the reduced model. Signaling through PI3K and PIP3 affects both commonly recognized downstream targets, such as protein kinase B, and important distinct pathways such as those containing ERK and p53 [44]. A recent mapping of the complete ErbB signaling network reveals PIP3 and its upstream kinase PI3K as highly informative nodes upon which a large fraction of signaling information converges [45]. Not surprisingly, then, we identify four proteins in our network gauge that interact with or are downstream of PIP3 or PI3K. These molecules are: Shc, SHIP-2, TfR, and SCF38 [22,37,46]. Thus, model reduction not only identifies a network gauge, but also suggests salient elements of the signaling network.

The PLSR model's ability to predict levels of proliferation and migration in 24H cells given only data from parental cells indicates that, although signals drastically change as we move from parental to 24H cells, the cell decides upon levels of migration and proliferation according to the same “rules.” These rules are nonintuitive but amount to the calculation of behavior according to the regression equation given by the PLSR model. Identification of conserved algorithms used to control behavior across cell type highlights the potential to predict a priori how changes in signaling will affect cell behavior and gives insight into conserved themes for cellular decision-making processes. Thus, the linear mapping of phospho-proteomic data onto cellular phenotype identified a key set of signals descriptive and predictive of phenotype in breast epithelial cells. It also identified subsets of signals that govern phenotype under either ligand or receptor perturbation, and in that process revealed new hypotheses about HER2-mediated signaling events. Of course, these hypotheses need to be tested through further focused molecular and biochemical work. Nevertheless, the modeling approach we introduce here is a powerful first step toward understanding signaling networks and the behaviors they control.

## Methods

### Mass spectrometry.

Samples were analyzed using mass spectrometry as previously described [10,47].

### Cell migration.

Migration was assayed as previously described [10,48]. Briefly, a wound healing analysis provided rates of wound closure. Error bars represent the 95% confidence intervals for the fit of the slope using linear regression.

### Cell proliferation.

Proliferation was assayed as previously described [10]. Briefly, a thymidine incorporation assay was used to measure proliferation 25 h after ligand stimulus. Error bars represent the standard deviation from four different biological replicates for each condition.

### Partial Least Squares Regression.

The PLSR model was generated using a SIMCA-P (10.0) software package as described elsewhere [10]. Briefly, an MxN data matrix (**X**) was generated from the mass spectrometry signaling dataset. Each column corresponded to one of the following four metrics: protein phosphorylation at 5 min, 10 min, 30 min, or the integral of protein phosphorylation from 0–30 min, used as a proxy for the total amount of protein phosphorylation during the 30-min duration. There were 248 columns in total, corresponding to the four metrics × 62 phosphosites. Each row represented a different cellular condition, with a total of six rows corresponding to parental serum-free, parental plus EGF (100 ng/mL), parental plus HRG (80 ng/ml), 24H serum-free, 24H plus EGF (100 ng/ml), and 24H plus HRG (80 ng/mL). An MxP matrix (**Y**) was generated from the cellular output data, with the rows corresponding to the same cellular conditions listed above and the columns representing cell migration and cell proliferation. All data were mean-centered and scaled to unit variance.

PLSR was used to solve the regression problem:

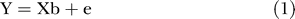
where **b** is the vector containing the regression coefficients and **e** is the residual. A nonlinear iterative partial least squares (NIPALS) algorithm was used [49,50]. It is instructive to note that the NIPALS algorithm applied to a single MxN matrix (**X**) is the representation of the matrix as a sum of outer products such that:

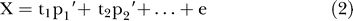
where t_i_ is called the scores vector and represents one dimension in the orthogonal basis set for the column space, and p_i_ is called the loadings vector and represents one dimension in the orthogonal basis set for the row space. Application of NIPALS in this way is analogous to the singular value decomposition of the matrix such that:

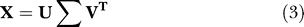
where each scores vector is equivalent to a column of the product *U*Σ, and each loadings vector is equivalent to a column of **V**.

The NIPALS algorithm was implemented as described elsewhere [50]. Briefly, an iterative process is used to define two vectors, w and c, that maximize the following term:

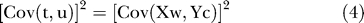
where t and u are the scores vectors for the **X** and **Y** matrices, respectively. Loadings vectors for **X** and **Y**, called p and q respectively, are also defined as:

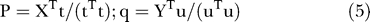



The PLS regression vector b' is defined as:

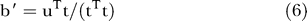



The set of vectors t,u,w, and c are associated with the maximum eigenvalues for various covariance matrices, and once defined, their contribution is removed from the **X** and **Y** matrices, leaving a residual matrix that can be further modeled with a new set of t,u,w,c,p, and q vectors. The matrices corresponding to each of these vectors are defined as **T**,**U**,**W**,**C**,**P**, and** Q**. The residual matrices are defined as:

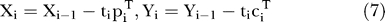
where the maximum value of i (referred to as A later for clarity) depends on the results of cross-validation. Weights were defined as w^*^, which are calculated from w to relate to the original X-matrix (and not the residual as calculated above) as:

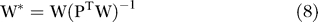



Each model was tested for goodness of prediction (Q^2^) using a leave-one-out cross-validation approach [51]. Briefly, cross-validation is performed by omitting an observation from the model development and then using the model to predict the **Y**-matrix values for the withheld observation. This procedure is repeated until every observation has been kept out exactly once. The prediction error sum of squares (PRESS) is then calculated as:

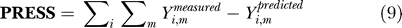



Q^2^ is then calculated as:

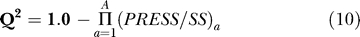
where *a* refers to each individual principal component in the model and *SS* is the sum of squares explained by principal component *a*. Standard errors and confidence intervals (as evaluated for the models presented) can be derived from cross-validation as well [51].

To evaluate the scores plot transitions (Tables 1–6), the vector **T_j,1:A_** – **T_k,1:A_** was evaluated to represent the transition from the cellular condition described by row k of **T** to the cellular condition represented in row j of **T**. The inner product of this vector and the weight of variable m (W^*^_m,1:A_) was evaluated for all variables (m = 1:248) and then ranked by magnitude.

To identify the signaling metrics most important for the overall model, a weighted sum of squares (also known as the variable importance for projection [VIP]) value for each variable (k) was calculated according to the following formula [51]:

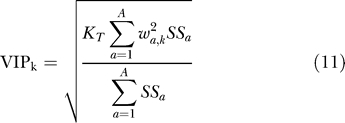
where K_T_ is the total number of variables and the rest of the variables are as described above. Signals having multiple metrics that ranked in the top 30 highest VIP scores were chosen for the reduced model.

To evaluate the importance of a given signal for an output, the inner product of each metric with a given output was evaluated by:

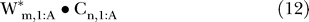
for all variables (m = 1:248) for each of both outputs (n = 1:2). To calculate the unique contribution of the signaling metrics to a given output (say n = 1), we evaluated the following expression:

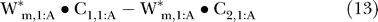
for all m. Overall contributions to the model were calculated as:

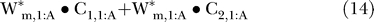



## Supporting Information 

### 

Dataset S1 All Scores Transitions Not Listed in the Main Text or in Table S1 Are Given Here The listed metrics are ranked from most positively correlated to negatively correlated for the indicated transition. A table of abbreviations including accession number, residue, and sequence is also provided in Table S2.
(63 KB XLS)

Figure S1 A Linear Model Accurately Recapitulates Experimental Data Results from PLSR modeling show that a computational model based on experimental data generates predicted values of cellular migration and proliferation that correlate strongly with experimentally measured values. Experimental values of migration (A) or proliferation (B) are graphed along the ordinate, and model predictions of migration (A) or proliferation (B) are given along the abscissa. R^2^ values indicate the data's goodness of fit to the line y = x. Experimental error bars denote 95% confidence intervals for migration (see Methods) and ± standard deviation for proliferation. All computational error bars are calculated from jack-knifing as a standard error.
(217 KB PDF)

Figure S2 A Linear Model Based Only on Parental Cell Data Predicts 24H Proliferation and Migration A PLSR model constructed from parental cell data only was used to predict (A) proliferation and (B) migration levels in 24H cells and then compared with measured experimental values. Experimental error is denoted by 95% confidence intervals for cell migration (see Methods) and ± standard deviation for cell proliferation. Computational error bars were calculated as the 95% confidence intervals based on jack-knifing.
(205 KB PDF)

Table S1 Signaling Metrics Most Important for Changes in Cellular Response due to Changes in HER2 Expression under Serum-Free Conditions Analyzed results from PLSR-generated scores plots reveal the 20 signaling metrics most positively correlated with cell behavior as HER2 levels are increased.
(38 KB DOC)

Table S2 Accession Numbers and Abbreviations for the Mass Spectometry Dataset (63 KB XLS)

### Accession Numbers

Accession numbers and further protein information are listed in Table S2.

## Abbreviations

ACK: activated cdc42-associated kinase
Dsc3a: desmocollin-3
EGF: epidermal growth factor
EGFR: epidermal growth factor receptor
ephA2: ephrin A2 receptor
ERK1: extracellular regulated kinase 1
FAK: focal adhesion kinase
GRF1: glucocorticoid receptor DNA binding factor 1
HRG: heregulin
HER: human epidermal growth factor receptor
HMEC: human mammary epithelial cells
MEK: mitogen-activated protein extracellular kinase
NIPALS: nonlinear iterative partial least squares
PI3K: phosphoinositide 3-kinase
PLSR: partial least squares regression
PIP3: phosphatidylinositol-3,4,5-trisphosphate
PRP4K: serine/threonine protein kinase PRP4 homolog
PTPRA: protein tyrosine phosphatase receptor type A
SCF38: solute carrier protein 38
Shc: SH2-containing protein
SHIP-2: SH2-containing inositol polyphosphate 5-phosphatase
TfR: human transferrin receptor
TGF-α: transforming growth factor alpha
VIP: variable importance for projection
