# Supplementary material for: Modeling HER2 Effects on Cell Behavior from Mass Spectrometry Phosphotyrosine Data
Source: PLoS Comput Biol. 2007 Jan 5;3(1):e4. doi: 10.1371/journal.pcbi.0030004 (PMC1761044; doi:10.1371/journal.pcbi.0030004)
Supplement: Supplementary file 1 [file pcbi.0030004.sg001.pdf]

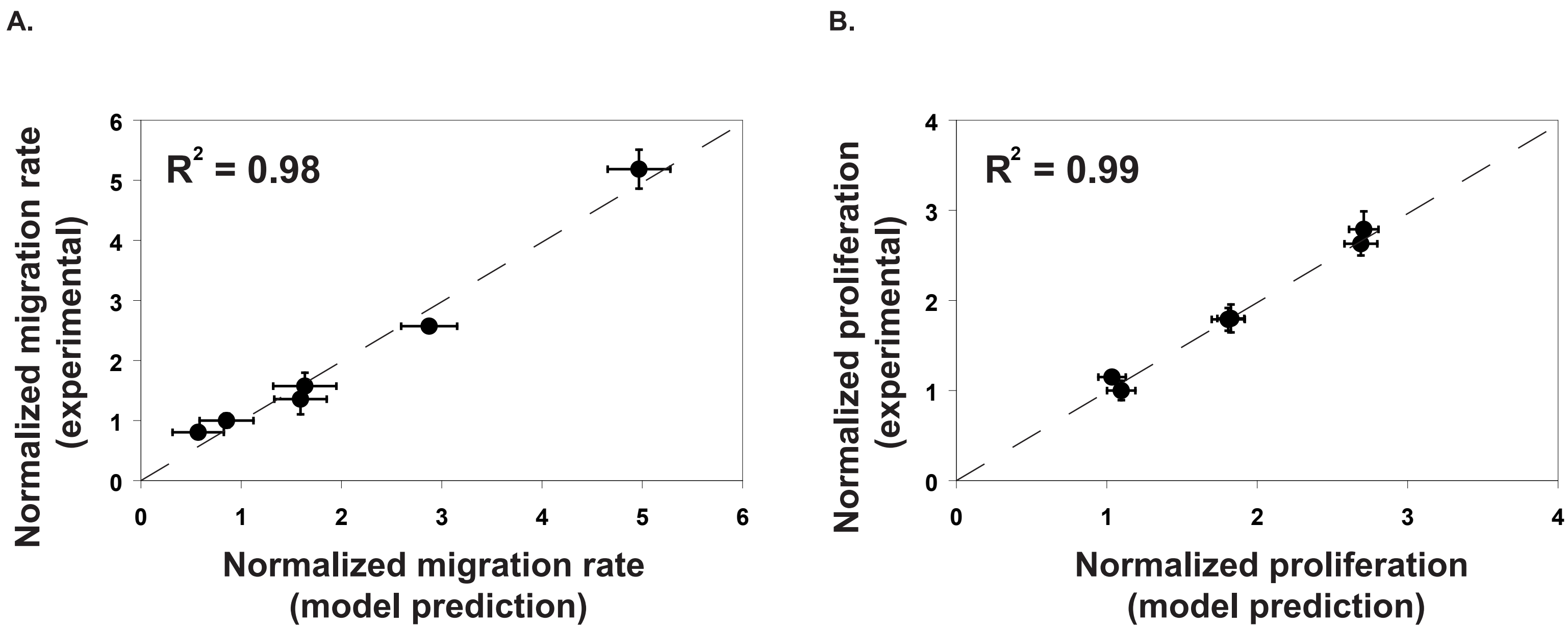
A.
Normalized migration rate (experimental)
R² = 0.98
Normalized migration rate (model prediction)
0
1
2
3
4
5
6
B.
Normalized proliferation (experimental)
R² = 0.99
Normalized proliferation (model prediction)
0
1
2
3
4
