# Supplementary material for: Modeling HER2 Effects on Cell Behavior from Mass Spectrometry Phosphotyrosine Data
Source: PLoS Comput Biol. 2007 Jan 5;3(1):e4. doi: 10.1371/journal.pcbi.0030004 (PMC1761044; doi:10.1371/journal.pcbi.0030004)
Supplement: Supplementary file 2 [file pcbi.0030004.sg002.pdf]

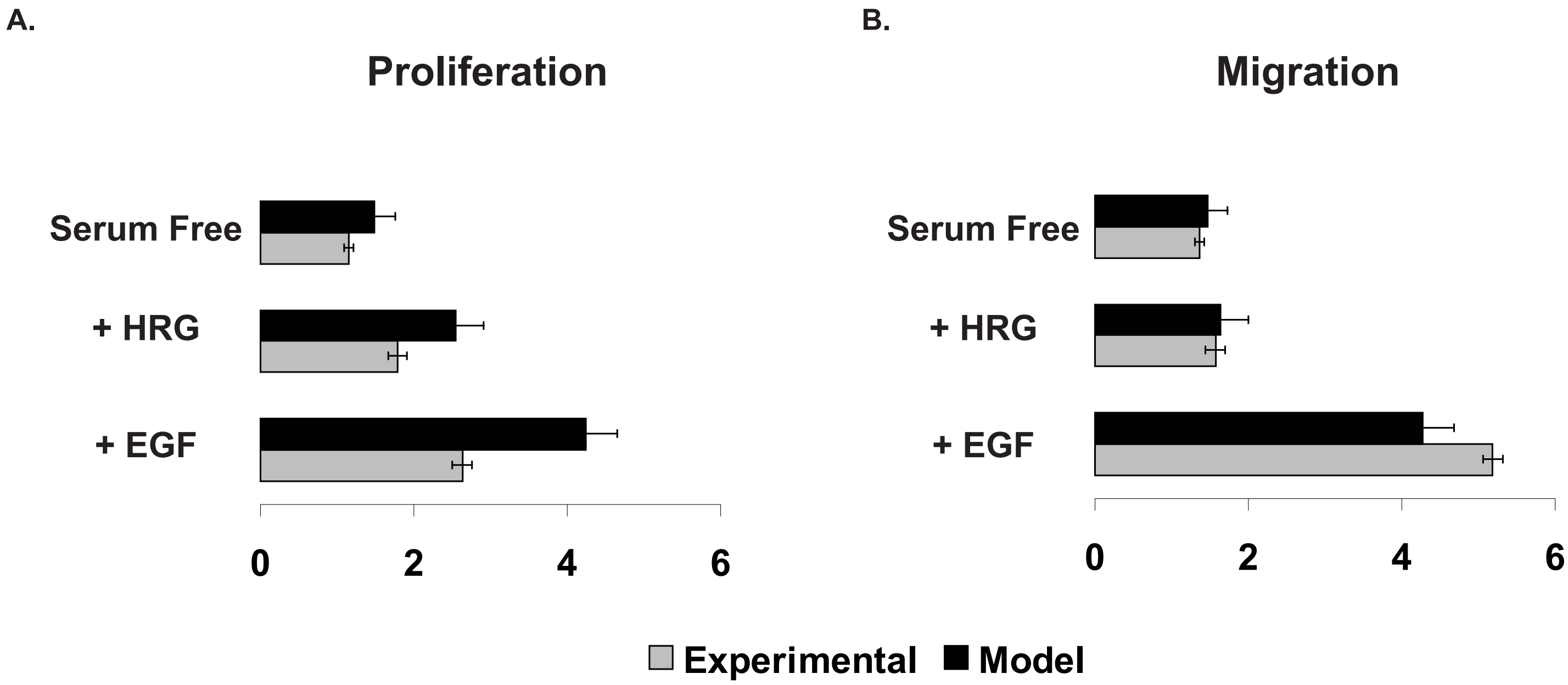
A.
Proliferation
Serum Free
+ HRG
+ EGF
0
2
4
6
B.
Migration
Serum Free
+ HRG
+ EGF
0
2
4
6
Experimental
Model
