# Supplementary material for: Modeling HER2 Effects on Cell Behavior from Mass Spectrometry Phosphotyrosine Data
Source: PLoS Comput Biol. 2007 Jan 5;3(1):e4. doi: 10.1371/journal.pcbi.0030004 (PMC1761044; doi:10.1371/journal.pcbi.0030004)
Supplement: Supplementary file 3 [file pcbi.0030004.st001.doc]

**Table S1**

| **Phosphorylation Site**  **(P→24H, SF)** | **Measurement**  **Type** |
| --- | --- |
| p130Cas Y 327 | 10 minutes |
| p130Cas Y 327 | 30 minutes |
| PRP4K Y 849 | 30 minutes |
| PTPRA Y 798 | 30 minutes |
| p130Cas Y 387 | 10 minutes |
| PTPRA Y 798 | 5 minutes |
| PTPRA Y 798 | 10 minutes |
| PI3KR2 Y 464 | 30 minutes |
| p130Cas Y 234 | 10 minutes |
| EphA2 Y/Y 588/594 | 5 minutes |
| SHP-2 Y 62 | 5 minutes |
| SHP-2 Y 62 | 10 minutes |
| PRP4K Y 849 | 5 minutes |
| SHP-2 Y 62 | 30 minutes |
| EphA2 Y 575 | 10 minutes |
| HER2 Y 1248 | 30 minutes |
| EphA2 Y 588 | 30 minutes |
| LDLR Y 845 | 30 minutes |
| p130Cas Y 387 | 30 minutes |
| LDLR Y 845 | 5 minutes |
